# Clinical diagnostic biomarker “circulating tumor cells” in breast cancer - a meta-analysis

**DOI:** 10.3389/fonc.2023.1137519

**Published:** 2023-06-16

**Authors:** Shiyan Bai, Shujin Lin, Ting Lin, Qiaowen Wang, Cui Cheng, Junru Lin, Ying Zhang, Xiwen Jiang, Xiao Han

**Affiliations:** ^1^ College of Biological Science and Engineering, Fuzhou University, Fuzhou, China; ^2^ Industrial Management Engineering, National University of Singapore, Singapore, Singapore

**Keywords:** circulating tumor cells, breast cancer, diagnostic, meta-analysis, liquid biopsy

## Abstract

**Objective:**

Using meta-analysis, we evaluate circulating tumor cells(CTCs) as a potential diagnostic tool for breast cancer.

**Methods:**

A document search was conducted using publicly available databases up to May 2021. Specific inclusion and exclusion criteria were formulated and summarize relevant data through literature types, research types, case populations, samples, etc. Subgroup analysis of documents based on regions, enrichment methods, and detection methods. The included research projects were evaluated using DeeKs’ bias, and evaluation indicators such as specificity (SPE), sensitivity (SEN), diagnosis odds ratio (DOR) were used as evaluation indicators.

**Results:**

16 studies on the use of circulating tumor cells to diagnose breast cancer were included in our meta-analysis. Overall sensitivity value was 0.50 (95%CI:0.48-0.52), specificity value was 0.93 (95%CI:0.92- 0.95), DOR value was 33.41 (95%CI:12.47-89.51), and AUC value was 0.8129.

**Conclusion:**

In meta-regressions and subgroup analysis, potential heterogeneity factors were analyzed, but the source of heterogeneity is still unclear. CTCs, as a novel tumor marker, have a good diagnostic value, but its enrichment and detection methods still need to continue to be developed to improve detection accuracy. Therefore, CTCs can be used as an auxiliary means of early detection, which is helpful to the diagnosis and screening of breast cancer.

## 1 Introduction

Globally, breast cancer is the second most common type of cancer, especially among women (1). The key to successful treatment of breast cancer is early diagnosis, which also reduces mortality (2). The gold standard for imaging breast cancer is mammography, but due to its limitations on dense breast tissue, additional detection methods are needed. A complementary detection method commonly used is ultrasound. A limitation of ultrasound is that it may not be able to detect microcalcifications and may miss early signs of tumors (3). To detect breast cancer early, simple, fast, and cost-effective blood based biomarkers are necessary, and these biomarkers can be used alongside mammography.

The liquid biopsy is a minimally invasive and harmless detection method. This low-invasive sampling uses circulating biomarkers to analyze tumors and provide reliable diagnosis. Liquid biopsy contains exosomes (4),microRNA(miRNA) (5), circulating tumor DNA(CtDNA) (6), circulating tumor cells (CTCs) (7), etc. Circulating tumor cells are those that become detached from the primary tumor and enter the peripheral circulation. Some of the cells escape the body’s immune system and undergo epithelial mesenchyme. The transformation and other processes are highly invasive, including tumor DNA information, the genome and proteome information, and can dynamically monitor tumor activity. A metastatic breast cancer patient’s blood contained CTCs during autopsies performed by Thomas R. Ashworth (8). Since then, more and more studies have used CTCs as breast cancer biomarkers to diagnose the disease (9). Therefore, this meta-analysis, through statistical research on the use of CTCs to detect early breast cancer, can draw the reliability of CTCs as early diagnostic markers of early breast cancer.

## 2 Materials and methods

### 2.1 Search strategy

Two authors independently searched Embase, PubMed, Elsevier Science Direct, and Medline databases for all potentially relevant articles published from 2004 to May 2021. The query terms were as follows:”CTCs” or “circulating tumor cell” and “breast carcinoma” or “breast cancer” or “mammary cancer” or “mammary carcinoma” and “diagnosis”. We also manually screened references in related articles to expand the scope of the search.

### 2.2 Criteria for inclusion and exclusion

Initially, two authors independently and rigorously evaluated our inclusion criteria, and those with differences were decided through consultation. Inclusion criteria: 1. Research type: a publicly published diagnostic test on the accuracy of circulating tumor cells in the diagnosis of breast cancer; 2. The case group is breast cancer patients diagnosed by the gold standard (pathological results); 3. Blood is the only study Sample standard. At the same time, using the exclusion criteria to eliminate the included documents, 1. Exclude animal experiments from basic research; 2. Exclude reviews, conference papers, abstracts, lectures, reviews and case reports; 3. Exclude documents whose four tables cannot be extracted 4. In the diagnosis of breast cancer, the circulating tumor cell index is not used; 5. Exclude the repeated publication of the literature; 6. Exclude the literature of combined diagnosis with other tumor markers.

### 2.3 Data extraction

Two authors independently extracted the relevant data needed for meta-analysis from the included articles, including basic information of the articles (first author, research year, region); research objects (number of people, disease types, age); research details (enriched Methods, diagnostic methods, thresholds, target indicators, sample source; surface antigen detection); Diagnostic indicators (specificity(SPE), sensitivity(SEN), and diagnosis odds ratio(DOR)), and further summarize the data. For the missing diagnostic indicators in the article, we use revman’s calculator for conversion calculations. Missing data that does not affect the accuracy of the meta-analysis, we tried to contact the author by email to obtain it, but the complete data was not obtained, so we chose to leave this item blank.

### 2.4 Quality assessment

The included literature was independently assessed by two authors using QUADAS-2 in Revman software. Different opinions on the quality evaluation were reached through discussion before the quality evaluation. The evaluation involved the selection of cases, the evaluation of the test, the gold standard, as well as the case process. And the progress, the iconic issues are included in the judgment of the risk of bias. The identifying issues in the design of QUADAS-2 are related to the potential of bias and are designed to help the evaluator judge the risk of bias.

### 2.5 Statistical analysis

In the meta-analysis, revman, stata, and MetaDisc software were used. Evaluation indicators were positive likelihood ratios, sensitivity, specificity, negative likelihood ratios, and diagnosis odds ratios. To evaluate the threshold effect, Spearman correlation is used, and Q and I² statistics are used to detect whether there is heterogeneity or the degree of heterogeneity. Spearman coefficient clarifies whether there is a threshold effect, and Spearman’s strong positive correlation shows that threshold effect exists. I^2^ of 0% suggests that no heterogeneity is observed, and the larger the value, the greater the heterogeneity. Significant Q value and I^2^>50% indicate that the included studies are heterogeneous, a random effects model is needed, and the sources of heterogeneity are explored by meta-regression and subgroup analysis, and publication bias is assessed by Deeks’ test.

## 3 Result

### 3.1 Document characteristics

**Figure 1:**
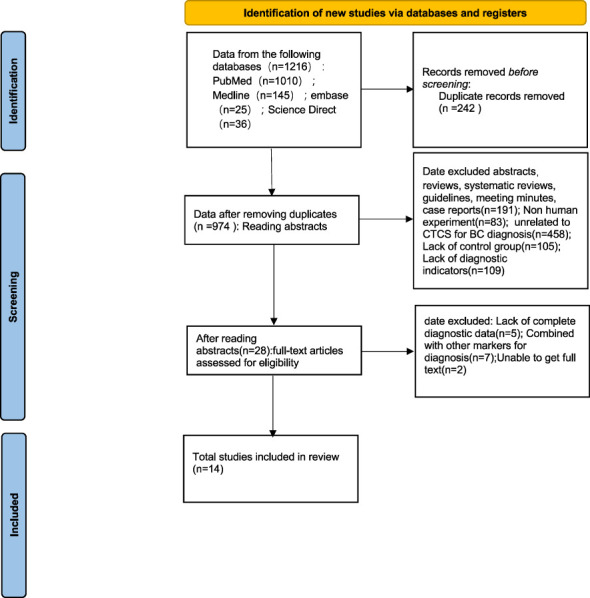
Flow chart of studies inclusion and screening.

We searched a total of 1216 articles from embase, PubMed, Elsevier Science Direct, and Medline databases, of which 14 articles (10–23) met our inclusion requirements, including sixteen studies with 2860 breast cancer patients and 1958 controls, including healthy individuals and patients with benign breast disease (**Figure 1**, **Table 1**). Seven of these studies are based on the principle of immunomagnetic beads to enrich CTCs, and seven of these studies are based on the principle of microfluidic chip to enrich CTCs. Four studies have used EpCAM as a target for enrichment detection.

**Table 1:** Details of included articles.

author	Region	enriched methods	identification	cutoff	BC patients	other patients	healthy females	control type	Sample source	TP	FP	FN	TN
chen 2018 (10)	china	LiquidBiopsy	immunofluorescence	1cell/4ml	71	NA	107	NA	peripheral blood	29	40	42	67
Yagata H 2008 (11)	Japan	cellsearch	CellSpotter	2cell/7.5ml	38	30	57	MBC	Blood samples	19	1	19	86
Park HS 2017 (12)	Korea	/	RT−PCR	Ct≥36	126	116	30	MBC	peripheral blood	132	0	113	30
jin 2011 (13)	Japan	OBP-401	fluorescence mounting medium	1cell/7.5ml	50	27	80	MBC、EBC	peripheral blood	25	0	52	80
jin 2011 (13)	Japan	cellsearch	fluorescence mounting medium	5cell/7.5ml	NA	NA	NA	NA	peripheral blood	43	0	34	80
jin 2020 (14)	china	CytoSorter ^®^	immunofluorescence staining	2cell/4ml	130	236	30	NA	peripheral blood	98	12	30	249
L Sanislo 2010 (15)	/	polycarbonate membrane	laser scanning cytometry	2cell/9ml	13	3	12	BC、MBC	peripheral blood	10	1	6	11
li 2017 (16)	china	Cellcollector	immunocytochemistry	1cell	127	67	/	MBC	peripheral blood	95	0	32	63
liu 2018 (17)	china	microfluidic chip	Three-color immunocytochemistry	1cell/2ml	20	NA	15	BC	whole blood	13	1	7	14
lu 2018 (18)	china	wedge-shaped microfluidic device	Three-color immunocytochemistry	1cell/2ml	10	23	25	NA	peripheral blood	8	1	2	9
Sawada T 2016 (19)	Japan	FCMC	immunostaining solution	3cell/1.6ml	22	NA	20	BC	peripheral blood	17	9	5	11
Allard WJ 2004 (20)		cellsearch	NA	2cell/7.5ml	422	199	145	NA	peripheral blood	489	1	827	343
yang 2021 (21)	china	CytoSorter^®^	immunofluorescence microscopy	2cell/4ml	238	217	20	/	peripheral blood	174	19	64	218
yuan 2015 (22)	china	iFISH	Cytelligen	NA	45	NA	14	BC	peripheral blood	41	0	4	10
yuan 2015 (22)	china	cellsearch	Cytelligen	NA	45	NA	14	BC	peripheral blood	17	0	28	10
zhang 2020 (23)	china	PDMS	immunofluorescence staining	3.5cell/ml	129	NA	50	/	peripheral blood	95	9	34	41

NA, not available; MBC, metastatic breast cancer; EBC, early breast cancer; BC, breast cancer.

### 3.2 Threshold effect

One of the major sources of heterogeneity in diagnostic meta-analysis is the presence of threshold effect, which should be checked as the first step. To detect the threshold effect, the Spearman correlation coefficient was calculated between sensitivity and specificity. A strong negative correlation with *p* < 0.05 implies the existence of a threshold effect. In our study, t the correlation coefficient R-value was 0.482 and the p-value was 0.059, indicating the absence of threshold effect (**Table 2**).

**Table 2:** Analysis of diagnostic threshold.

Var	Coeff.	Std. Error	T	p-value
a	2.167	0.521	4.157	0.001
b(1)	-0.533	0.177	3.02	0.0092

Tau-squared estimate = 1.0598 (Convergence is achieved after 15 iterations).

Spearman correlation coefficient: 0.482 p-value= 0.059.

Moses’ model (D = a + bS).

No. studies = 16.

### 3.3 The overall study of CTC diagnosis of BC

**Figure 2:**
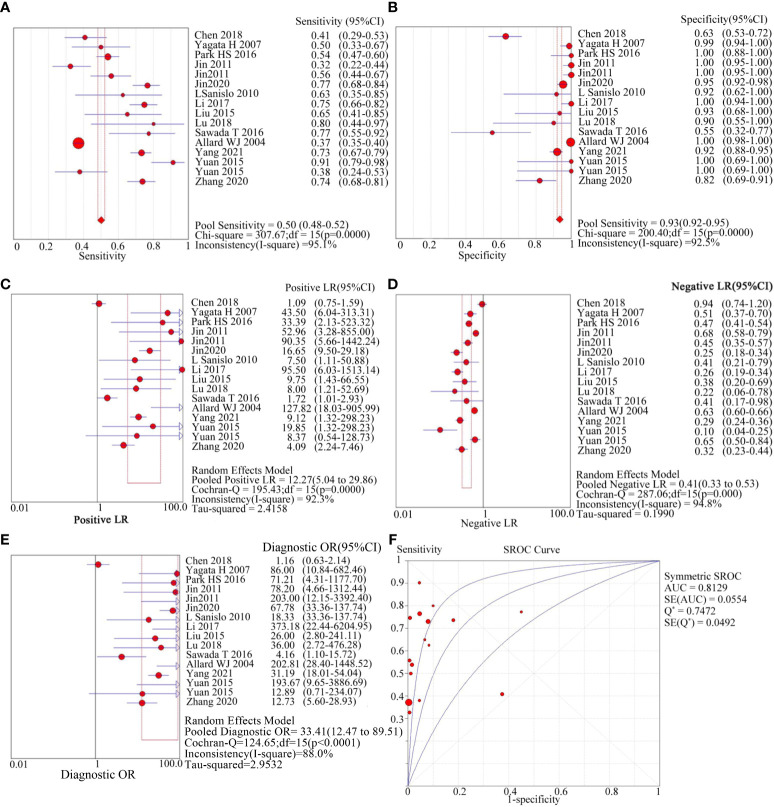
diagnostic value of circulating tumor cells for breast cancer patients. **(A)**: forest plot of sensitivity of circulating tumor cells for diagnosis of breast cancer patients; **(B)**: forest plot of specificity of circulating tumor cells for diagnosis of breast cancer patients; **(C)**: forest plot of positive likelihood ratio of circulating tumor cells for diagnosis of breast cancer patients; **(D)**: forest plot of negative likelihood ratio of circulating tumor cells for diagnosis of breast cancer patients; e: forest plot of circulating tumor cells for breast cancer patients; **(E)**: forest plot of the diagnostic advantage ratio of circulating tumor cells to breast cancer patients; **(F)**: receiver operating characteristic curve(ROC) of circulating tumor cells to breast cancer patients.

The meta-analysis included 1544 patients from 16 studies across 14 articles. It involved recording the number of true/false positives and negatives as well as calculating the negative likelihood ratio (LR-), positive likelihood ratio (LR+), specificity (SPE), sensitivity (SEN), diagnostic odds ratio (DOR), and summary receiver operating characteristic (SROC) curve. **Figure 2** shows the results: SEN was 0.50 (95%CI=0.48-0.52), SPE was 0.93 (95%CI=0.92-0.95), LR+ was 12.27 (95%CI=5.04-29.86), LR- was 0.41 (95%CI=0.33-0.53), DOR was 33.41 (95%CI=12.47-89.51), AUC was 0.8129, and Q* was 0.7472. However, there was a large heterogeneity (I^2 ^= 95.1% for SEN and 92.5% for SPE) as shown in **Table 3**, indicating that further investigation is needed to explore the source of heterogeneity.

**Table 3:** Overall research analysis.

Parameter	Estimate	95%CI	Q	*p*	I^2^%	T^2^	Model
Sensitive	0.5	0.48-0.52		< 0.01	95.1		
Specificity	0.93	0.92-0.95		< 0.01	92.5		
Positive LR	12.27	5.04-29.86	195.43	< 0.01	92.3	2.4158	Random
Negative LR	0.41	0.33-0.53	287.06	< 0.01	94.3	0.199	Random
Diagnostic Odds Radio	33.41	12.47-89.51	124.65	< 0.01	88	2.9532	Random

### 3.4 Analyze the sources of heterogeneity

#### 3.4.1 Exploring the sources of heterogeneity with meta-regression

**Tables 4**–**7** shows the results of the meta-regression analysis conducted to explore the effects of region, literature quality, and assay method on heterogeneity. The analysis found that different methods of enriching circulating tumor cells were the main source of heterogeneity (RDOR = 0.08, 95% CI = 0.04-0.16), while region, literature quality, and assay method had no significant effect.

**Table 4:** Meta-Regression (Inverse Variance weights).

Var	Coeff.	Std.Err.	p-value	RDOR	95%CI
Cte.	3.474	0.9977	0.0059	—	—
S	-0.895	0.1765	0.0005	—	—
enriched	-2.24	0.4801	0.0009	0.11	(0.04-0.31)
qualification	0.231	0.2531	0.3829	1.26	(0.72-2.21)
identification	0.335	0.6062	0.5931	1.4	(0.36-5.39)
region	0.321	0.4222	0.465	1.38	(0.54-3.53)

**Table 5:** Meta-Regression.

Var	Coeff.	Std.Err.	p-value	RDOR	95%CI
Cte.	3.768	0.8436	0.001	—	—
S	-0.911	0.1738	0.0003	—	—
enriched	-2.241	0.4801	0.0007	0.11	(0.04-0.31)
qualification	0.221	0.2525	0.3994	1.25	(0.72-2.18)
region	0.362	0.4155	0.402	1.44	(0.58-3.58)

**Table 6:** Meta-Regression.

Var	Coeff.	Std.Err.	p-value	RDOR	95%CI
Cte.	4.167	0.7085	0.0001	—	—
S	-0.885	0.1713	0.0002	—	—
enriched	-2.228	0.4799	0.0006	0.11	(0.04-0.31)
qualification	0.220	0.2525	0.3999	1.25	(0.72-2.16)

**Table 7:** Meta-Regression.

Var	Coeff.	Std. Err.	p - value	RDOR	95%CI
Cte.	4.646	0.4484	0	—	—
S	-0.993	0.1183	0	—	—
enriched	-2.531	0.3312	0	0.08	(0.04;0.16)

#### 3.4.2 Subgroup analysis

**Figure 3:**
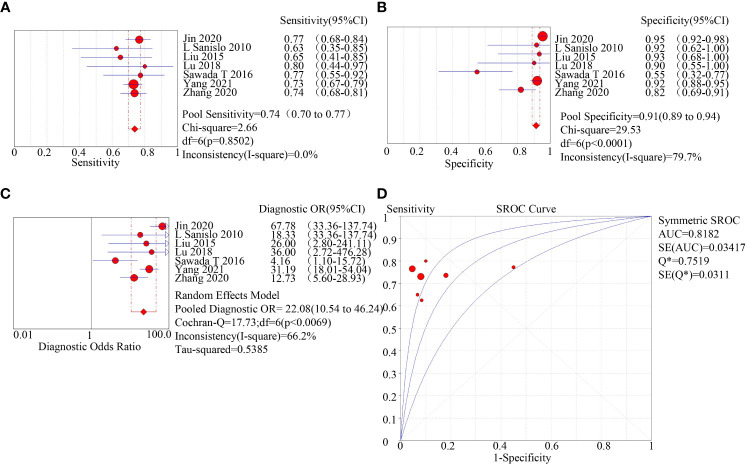
subgroup analysis of microfluidic chip enriched with CTC. **(A)**: forest plot of sensitivity of microfluidic chip enriched with CTC; **(B)**: forest plot of specificity of microfluidic chip enriched with CTC; **(C)**: forest plot of diagnostic advantage ratio of microfluidic chip enriched with CTC; **(D)**: receiver operating characteristic curve (ROC) of microfluidic chip enriched with CTC.

**Figure 4:**
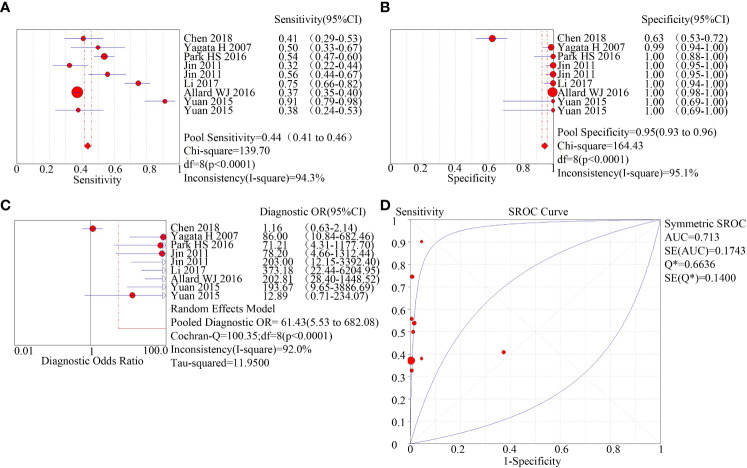
subgroup analysis of immunomagnetic bead adsorption enriched CTC. **(A)**: forest plot of sensitivity of immunomagnetic bead adsorption enriched CTC; **(B)**: forest plot of specificity of immunomagnetic bead adsorption enriched CTC; **(C)**: forest plot of diagnostic advantage ratio of immunomagnetic bead adsorption enriched CTC; **(D)**: receiver operating characteristic curve (ROC) of immunomagnetic bead adsorption enriched CTC.

Two methods for the enrichment of circulating tumor cells were sub-group analyzed: immunomagnetic bead adsorption-based enrichment and microfluidic chip enrichment. The microfluidic chip enrichment demonstrated a sensitivity (SEN) of 0.74 (95% CI=0.70-0.77), specificity (SPE) of 0.91 (95% CI=0.89-0.94), and area under curve (AUC) of 0.8182 (**Figure 3**). The immunomagnetic bead adsorption-based enrichment, on the other hand, showed a SEN of 0.44 (95% CI=0.41-0.46), SPE of 0.95 (95% CI=0.93-0.96), and AUC of 0.713 (**Figure 4**).

### 3.5 Quality assessment

**Figure 5:**
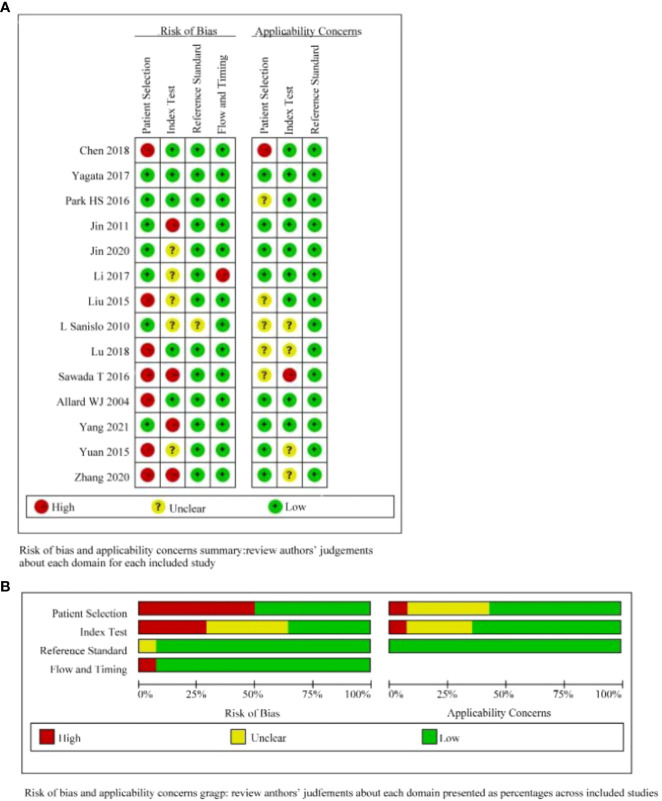
Quality evaluation of the included literature by QUADAS-2 based on Revman software. **(A)**:judgements about each domain for each included study; **(B)**: judgements about each domain presented as percentages across included studies.

We evaluated the quality of the 14 papers included in this study with the RevMan software (**Figure 5**). Most of the literature was of medium to high quality, suggesting that the included literature was reliable. All cases utilized pathological findings as the gold standard. Seven of the papers were susceptible to bias because they failed to avoid case-control studies. Six of the papers had an unknown risk because they did not specify whether the threshold was predetermined.

### 3.6 Publication bias

**Figure 6:**
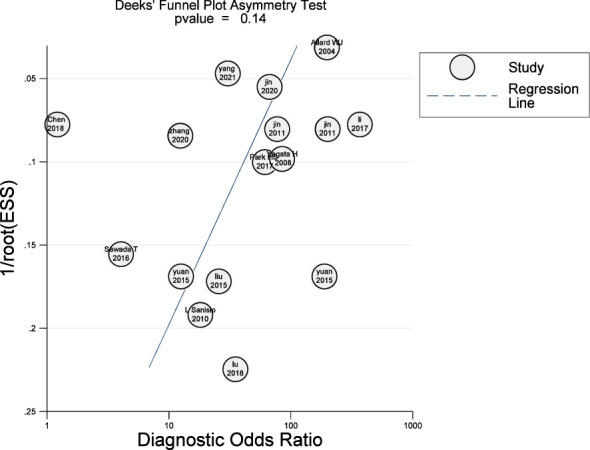
Deeks’ funnel plot asymmetry test to evaluate the publication bias of included studies.

We employed Deeks’ funnel plot asymmetry test to assess the possibility of publication bias among the studies considered in this analysis, by graphing the ratio of effect values to standard errors for each one. Sixteen studies were evaluated, all of which were drawn from 14 literatures. A significance level of *p* < 0.05 was taken to indicate the absence of publication bias within the literature surveyed. Our findings revealed that the surveyed literature was not affected by publication bias, as shown in **Figure 6**.

## 4 Discussion

Circulating tumor cells are cancer cells that are naturally shed from primary or metastatic tumors and circulate in the bloodstream, contributing to tumor metastasis (24). Timely diagnosis and treatment before metastasis are essential in reducing cancer-associated mortalities by 30%, as per the World Health Organization (25). Diagnostic confirmation of cancer usually involves invasive biopsies, including pathological analysis; however, these invasive procedures have limitations and may cause tumor spread, tissue damage, and organ damage (24). Although cancer may still be in its early stage, CTCs can be detected in the peripheral blood of cancer patients (26). Circulating tumor cells as a novel liquid biopsy technique for minimally invasive early tumor detection by detecting circulating tumor cells in the peripheral blood of tumor patients (27).

A meta-analysis was conducted to examine the diagnostic efficacy of using CTCs in breast cancer patients. The results revealed that the sensitivity of CTCs for detecting breast cancer was 0.50 (95% CI=0.48-0.52), and the specificity was 0.93 (95% CI=0.92-0.95), with an AUC of 0.8129. The diagnostic performance of CTCs can be assessed using positive and negative likelihood ratios. Our meta-analysis demonstrated that the positive likelihood ratio of CTCs in breast cancer diagnosis was 12.27 (95% CI=5.04-29.86), while the negative likelihood ratio was 0.41 (95% CI=0.33-0.53). These findings indicate that CTCs have a high diagnostic value in breast cancer.

Circulating tumor cells (CTCs) are sparse within the bloodstream (28). The efficient enrichment of CTCs is crucial in the diagnosis and treatment of tumors. However, identifying and enriching CTCs are the most challenging aspect of diagnosing diseases using liquid biopsy techniques. There are several methods available for enriching CTCs, such as the immunomagnetic bead method, microfluidic chip method, and filter membrane method. Among them, the immunomagnetic bead method is the most widely used. This method focuses on binding specifically to CTC surface markers including epithelial cell adhesion molecules (EpCAM) present on the surface of these cells. In contrast, the microfluidic chip is a liquid biopsy technique receiving increased recognition, that separates intact circulating tumor cells basing on their unique physical characteristics like size, deformability, density, hydrodynamic, and dielectric properties (29–31), avoiding the effect of ligand binding on cell membranes, and identifying specific identified circulating tumor cells based on physical properties in combination with biological methods such as antibodies and ligands (32). We conducted a meta-analysis to investigate the source of heterogeneity and found that it originated from the method of enrichment of circulating tumor cells. We included a total of 16 studies in our analysis, and subgroup analysis based on the method of enrichment revealed that the sensitivity (0.74 vs. 0.44) and area under the curve (AUC) of the microfluidic chip method of enrichment were higher than those of the immunomagnetic bead method (0.818 vs. 0.713). Enrichment of circulating tumor cells by microfluidic chip is considered one of the most promising methods for CTC isolation, detection, and downstream assays. Despite substantial advancements in these technologies over the last few decades, there remain several challenges such as reducing clogging, increasing enrichment efficiency, and ensuring high cell viability.

CellSearch is an FDA-approved method for enriching circulating tumor cells (CTCs) expressing EpCAM and CK using specific antibodies (33). However, CTCs are prone to epithelial mesenchymal transition (EMT), resulting in the loss of epithelial markers and overexpression of mesenchymal markers. As a result, CellSearch cannot detect CTCs expressing non-epithelial phenotypes. When used in combination with CellSpotter identification for breast cancer diagnosis by Yagata (11), Allard WJ (20) and Yuan et al (22) CellSearch demonstrated limited sensitivity. Probably because they are based on the phenotypic characteristics of circulating tumor cells for enrichment and detection analysis (34).In particular, CTCs are sometimes phenotypically similar to normal cells, in which case CellSpotter may misidentify normal cells as circulating tumor cells, thus reducing the accuracy of circulating tumor cell detection. Notably, Jin (14), Sawada (19) and Zhang (23) et al, used microfluidic chip enriched for circulating tumor cells combined with immunofluorescent staining to detect circulating tumor cells has greatly improved the diagnostic value of circulating tumor cells in breast cancer. Microfluidic chip with immunoassay technologies, as novel assays with great development prospects, are expected to play an increasingly important role in clinical oncology, providing strong support and guarantee for early diagnosis, precise treatment and prognosis assessment of tumors.

There were several limitations in our meta-analysis. First, in the included articles, due to incomplete data in some articles and incomplete data that are not sufficient to obtain the required data through data transformation, our meta-analysis cannot include it. In our meta-analysis, although heterogeneity was investigated by meta-regression, the meta-regression results showed that the source of heterogeneity was related to the enrichment method of circulating tumor cells, and the subgroup heterogeneity based on immunomagnetic bead enrichment of circulating tumor cells for breast cancer diagnosis by subgroup analysis was still high, which may be due to the fact that the authors used different immunological indicators for enrichment of circulating tumor cells in different studies.

## 5 Conclusion

By collecting relevant data from previous studies to meta-analysis, we found that CTCs as markers for tumor diagnosis are expected to provide more effective and accurate support for early clinical diagnosis of breast cancer, especially the CTCs enrichment technology based on microfluidic chip showed higher sensitivity and diagnostic accuracy in the early diagnosis of breast cancer patients. In conclusion, the current study shows that CTCs have great potential as non-invasive biomarkers for breast cancer diagnosis, however, the low level of circulating tumor cells in the blood is a major challenge for its detection, and the development of more sensitive microfluidic chip for enrichment of circulating tumor cells could improve the diagnostic accuracy of breast cancer.

## Data availability statement

The original contributions presented in the study are included in the article, further inquiries can be directed to the corresponding author/s.

## Author contributions

Conceptualization, SB; methodology, SL; software, SB and TL; validation, SL, QW and CC; data curation, YZ and JL; writing—original draft preparation, SB; writing—review and editing, SL; supervision, XH and XJ; All authors have read and agreed to the published version of the manuscript. 
